# Supplementary material for: Modeling glioblastoma heterogeneity as a dynamic network of cell states
Source: Mol Syst Biol. 2021 Sep 16;17(9):e10105. doi: 10.15252/msb.202010105 (PMC8444284; doi:10.15252/msb.202010105)
Supplement: Supplementary file 6 — Source Data for Figure 5 [file MSB-17-e10105-s004.zip › Figure5A_sourcedata/GSEA_3017/nosigdb_stateC.GseaPreranked.1622795002785/RICHARDS_NOWAKOWSKI_SCIENCE_2017_MGE_RG2_UPREG.html]

Details for gene set RICHARDS\_NOWAKOWSKI\_SCIENCE\_2017\_MGE\_RG2\_UPREG[GSEA]

|  || Dataset | state13017 |
| Phenotype | NoPhenotypeAvailable |
| Upregulated in class | na\_pos |
| GeneSet | RICHARDS\_NOWAKOWSKI\_SCIENCE\_2017\_MGE\_RG2\_UPREG |
| Enrichment Score (ES) | 0.52759856 |
| Normalized Enrichment Score (NES) | 4.298939 |
| Nominal p-value | 0.0 |
| FDR q-value | 0.0 |
| FWER p-Value | 0.0 |
Table: GSEA Results Summary

  

Fig 1: Enrichment plot: RICHARDS\_NOWAKOWSKI\_SCIENCE\_2017\_MGE\_RG2\_UPREG      
 Profile of the Running ES Score & Positions of GeneSet Members on the Rank Ordered List

  

| PROBE | GENE SYMBOL | GENE\_TITLE | RANK IN GENE LIST | RANK METRIC SCORE | RUNNING ES | CORE ENRICHMENT || 1 | TFDP2 |  |  | 1 | 0.787 | 0.0196 | Yes |
| 2 | HES6 |  |  | 5 | 0.694 | 0.0326 | Yes |
| 3 | ATAD2 |  |  | 7 | 0.649 | 0.0484 | Yes |
| 4 | FAM111B |  |  | 8 | 0.616 | 0.0653 | Yes |
| 5 | CENPU |  |  | 10 | 0.588 | 0.0795 | Yes |
| 6 | HIST1H1C |  |  | 11 | 0.583 | 0.0955 | Yes |
| 7 | FAM111A |  |  | 13 | 0.576 | 0.1093 | Yes |
| 8 | CCNE2 |  |  | 15 | 0.570 | 0.1229 | Yes |
| 9 | CLSPN |  |  | 17 | 0.561 | 0.1363 | Yes |
| 10 | HMGB2 |  |  | 20 | 0.534 | 0.1470 | Yes |
| 11 | CDK6 |  |  | 24 | 0.518 | 0.1552 | Yes |
| 12 | CHAF1A |  |  | 27 | 0.494 | 0.1648 | Yes |
| 13 | ZBTB20 |  |  | 30 | 0.482 | 0.1740 | Yes |
| 14 | DLX1 |  |  | 32 | 0.469 | 0.1848 | Yes |
| 15 | GMNN |  |  | 38 | 0.453 | 0.1873 | Yes |
| 16 | ORC6 |  |  | 39 | 0.450 | 0.1996 | Yes |
| 17 | MMS22L |  |  | 40 | 0.448 | 0.2119 | Yes |
| 18 | ESCO2 |  |  | 43 | 0.440 | 0.2200 | Yes |
| 19 | UHRF1 |  |  | 45 | 0.436 | 0.2300 | Yes |
| 20 | RAD51AP1 |  |  | 46 | 0.435 | 0.2419 | Yes |
| 21 | PCNA |  |  | 48 | 0.435 | 0.2518 | Yes |
| 22 | TMPO |  |  | 49 | 0.432 | 0.2637 | Yes |
| 23 | DTL |  |  | 50 | 0.430 | 0.2755 | Yes |
| 24 | BRCA1 |  |  | 54 | 0.410 | 0.2808 | Yes |
| 25 | DHFR |  |  | 55 | 0.405 | 0.2919 | Yes |
| 26 | WDR76 |  |  | 56 | 0.403 | 0.3030 | Yes |
| 27 | TYMS |  |  | 60 | 0.390 | 0.3077 | Yes |
| 28 | RRM1 |  |  | 62 | 0.386 | 0.3163 | Yes |
| 29 | SMC2 |  |  | 63 | 0.386 | 0.3269 | Yes |
| 30 | GLCCI1 |  |  | 68 | 0.378 | 0.3292 | Yes |
| 31 | E2F7 |  |  | 69 | 0.377 | 0.3396 | Yes |
| 32 | DEK |  |  | 74 | 0.373 | 0.3418 | Yes |
| 33 | NASP |  |  | 75 | 0.371 | 0.3520 | Yes |
| 34 | POLD3 |  |  | 76 | 0.367 | 0.3621 | Yes |
| 35 | SMC4 |  |  | 78 | 0.365 | 0.3701 | Yes |
| 36 | ITGB3BP |  |  | 79 | 0.364 | 0.3801 | Yes |
| 37 | GINS2 |  |  | 83 | 0.360 | 0.3840 | Yes |
| 38 | BARD1 |  |  | 86 | 0.357 | 0.3898 | Yes |
| 39 | DUT |  |  | 87 | 0.356 | 0.3996 | Yes |
| 40 | MIS18BP1 |  |  | 89 | 0.355 | 0.4073 | Yes |
| 41 | MCM7 |  |  | 91 | 0.352 | 0.4150 | Yes |
| 42 | USP1 |  |  | 92 | 0.352 | 0.4246 | Yes |
| 43 | FOXN3 |  |  | 94 | 0.347 | 0.4321 | Yes |
| 44 | FEN1 |  |  | 98 | 0.342 | 0.4355 | Yes |
| 45 | NUSAP1 |  |  | 100 | 0.340 | 0.4429 | Yes |
| 46 | CKB |  |  | 102 | 0.335 | 0.4501 | Yes |
| 47 | NCAPD3 |  |  | 103 | 0.334 | 0.4592 | Yes |
| 48 | MCM3 |  |  | 114 | 0.324 | 0.4481 | Yes |
| 49 | TIMELESS |  |  | 119 | 0.319 | 0.4489 | Yes |
| 50 | HAT1 |  |  | 121 | 0.318 | 0.4556 | Yes |
| 51 | SMC1A |  |  | 125 | 0.312 | 0.4582 | Yes |
| 52 | HMGN2 |  |  | 126 | 0.312 | 0.4668 | Yes |
| 53 | DNMT1 |  |  | 127 | 0.311 | 0.4753 | Yes |
| 54 | STAG2 |  |  | 128 | 0.309 | 0.4838 | Yes |
| 55 | KIF15 |  |  | 136 | 0.302 | 0.4781 | Yes |
| 56 | RFC4 |  |  | 138 | 0.300 | 0.4844 | Yes |
| 57 | CENPK |  |  | 139 | 0.299 | 0.4926 | Yes |
| 58 | ASCL1 |  |  | 145 | 0.290 | 0.4905 | Yes |
| 59 | CCND2 |  |  | 149 | 0.288 | 0.4924 | Yes |
| 60 | RFC3 |  |  | 156 | 0.283 | 0.4882 | Yes |
| 61 | PARP1 |  |  | 163 | 0.280 | 0.4839 | Yes |
| 62 | H2AFY |  |  | 165 | 0.278 | 0.4896 | Yes |
| 63 | CHD7 |  |  | 166 | 0.277 | 0.4972 | Yes |
| 64 | VRK1 |  |  | 169 | 0.275 | 0.5007 | Yes |
| 65 | HELLS |  |  | 171 | 0.271 | 0.5062 | Yes |
| 66 | C19orf48 |  |  | 173 | 0.271 | 0.5116 | Yes |
| 67 | MAD2L1 |  |  | 174 | 0.270 | 0.5190 | Yes |
| 68 | RBBP8 |  |  | 177 | 0.267 | 0.5223 | Yes |
| 69 | POLA1 |  |  | 179 | 0.266 | 0.5276 | Yes |
| 70 | MCM4 |  |  | 189 | 0.259 | 0.5167 | No |
| 71 | CDK1 |  |  | 191 | 0.255 | 0.5217 | No |
| 72 | MTCL1 |  |  | 203 | -0.253 | 0.5067 | No |
| 73 | GPATCH4 |  |  | 220 | -0.258 | 0.4819 | No |
| 74 | ARL4A |  |  | 224 | -0.259 | 0.4830 | No |
| 75 | RPL27A |  |  | 228 | -0.259 | 0.4841 | No |
| 76 | CLIC1 |  |  | 237 | -0.261 | 0.4753 | No |
| 77 | RPL41 |  |  | 270 | -0.269 | 0.4188 | No |
| 78 | RPL23 |  |  | 288 | -0.273 | 0.3924 | No |
| 79 | RPS18 |  |  | 301 | -0.277 | 0.3760 | No |
| 80 | CREB5 |  |  | 309 | -0.281 | 0.3698 | No |
| 81 | RPL12 |  |  | 332 | -0.288 | 0.3338 | No |
| 82 | RPS8 |  |  | 371 | -0.307 | 0.2663 | No |
| 83 | PDPN |  |  | 372 | -0.307 | 0.2748 | No |
| 84 | FOS |  |  | 377 | -0.309 | 0.2753 | No |
| 85 | ZFP36L2 |  |  | 401 | -0.321 | 0.2381 | No |
| 86 | LDLR |  |  | 419 | -0.334 | 0.2134 | No |
| 87 | WWTR1 |  |  | 437 | -0.345 | 0.1889 | No |
| 88 | FAT1 |  |  | 441 | -0.346 | 0.1924 | No |
| 89 | HSPA5 |  |  | 447 | -0.350 | 0.1921 | No |
| 90 | BCAN |  |  | 449 | -0.351 | 0.1997 | No |
| 91 | LDHA |  |  | 498 | -0.412 | 0.1152 | No |
| 92 | OLIG2 |  |  | 499 | -0.415 | 0.1266 | No |
| 93 | SCD |  |  | 504 | -0.431 | 0.1304 | No |
| 94 | NES |  |  | 509 | -0.435 | 0.1344 | No |
| 95 | TNC |  |  | 564 | -0.572 | 0.0423 | No |
| 96 | CYR61 |  |  | 584 | -0.714 | 0.0240 | No |
Table: GSEA details [plain text format]

  

Fig 2: RICHARDS\_NOWAKOWSKI\_SCIENCE\_2017\_MGE\_RG2\_UPREG: Random ES distribution      
 Gene set null distribution of ES for **RICHARDS\_NOWAKOWSKI\_SCIENCE\_2017\_MGE\_RG2\_UPREG**
